# Hospital Sunday Collections in London in 1907

**Published:** 1908-02-01

**Authors:** 


### HOSPITAL SUNDAY COLLECTIONS IN LONDON IN 1907.

The *National Church* gives the following analysis of the collections made in the churches and chapels of the Metropolis last year. It will be seen that the contributions of the Church of England considerably exceed four-fifths of the whole amount collected :—

| | £ | s. | d. |
|---|---|---|---|
| Church of England ... ... | 34,473 | 1 | 3 |
| Congregationalists ... ... | 1,813 | 7 | 7 |
| Jews ... ... ... ... | 1,518 | 6 | 5 |
| Presbyterians ... ... ... | 1,112 | 3 | 7 |
| Wesleyans ... ... ... ... | 962 | 3 | 8 |
| Baptists ... ... ... ... | 830 | 8 | 11 |
| Roman Catholics ... ... ... | 705 | 16 | 3 |
| Unitarians ... ... ... | 366 | 13 | 0 |
| Church of Scotland ... ... | 205 | 8 | 6 |
| Catholic Apostolic ... ... | 98 | 13 | 3 |
| Greek Church ... ... ... | 87 | 2 | 6 |
| German Lutherans ... ... | 86 | 14 | 9 |
| Society of Friends ... ... | 75 | 10 | 1 |
| Foreign Protestants ... ... | 66 | 13 | 6 |
| Methodists (Welsh Calvinistic) | 40 | 14 | 6 |
| Reformed Episcopal Church ... | 30 | 14 | 6 |
| Methodists (United Free Church | 24 | 10 | 0 |
| Methodists (Primitive) ... ... | 17 | 13 | 3 |
| Swedenborgians ... ... ... | 17 | 11 | 10 |
| Free Church of England ... ... | 12 | 10 | 0 |
| Moravians ... ... ... ... | 2 | 16 | 8 |
| Methodists (New Connexion) ... | 1 | 1 | 0 |
| Various ... ... ... ... | 236 | 13 | 11 |
| | £42,786 | 8 | 11 |

The total sum raised (£42,786 8s. 11d.) is £1,848 less than the sum raised in 1906.

